# Implementation of Novel Lipid Therapies in a Refractory Heterozygous Familial Hypercholesterolemia Patient With Atherosclerotic Disease

**DOI:** 10.1016/j.jaccas.2022.06.021

**Published:** 2022-10-19

**Authors:** Spencer F. Weintraub, Jessica A. Schillow, Bani M. Azari, Benjamin J. Hirsh

**Affiliations:** a Zucker School of Medicine at Hofstra/Northwell, Department of Medicine, Northwell Health, Hempstead, New York, USA; b North Shore University Hospital, Northwell Health, Manhasset, New York, USA; c Zucker School of Medicine at Hofstra/Northwell, Department of Cardiology, Northwell Health, Hempstead, New York, USA

**Keywords:** atherosclerosis, dyslipidemias, hypercholesterolemia, lipid metabolism disorders, ACLY, adenosine triphosphate-citrate lyase, ANGPTL3, angiopoietin-like protein 3 inhibitor, ASCVD, atherosclerotic cardiovascular disease, FH, familial hypercholesterolemia, LDL-C, low-density lipoprotein cholesterol, LDL-R, low-density lipoprotein receptor, PCSK9, proprotein convertase subtilisin/kexin type 9

## Abstract

Compound heterozygous familial hypercholesterolemia patients are phenotypically similar to homozygous familial hypercholesterolemia patients, present with significant elevations of low-density lipoprotein cholesterol, and are at risk of cardiovascular disease. Although new treatment options are emerging, the stepwise approach to the use of different therapies has not been well described. (**Level of Difficulty: Intermediate.**)

## Central Illustration

**Figure:**
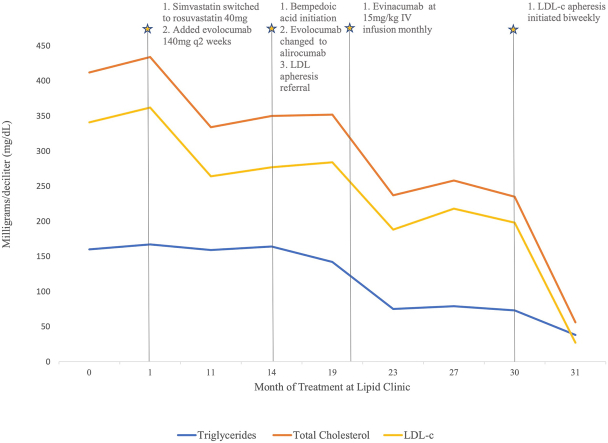

## History of Presentation

A 48-year-old woman presented to our Lipid Center for significantly elevated low-density lipoprotein cholesterol (LDL-C), despite medical treatment. Physical examination revealed vital signs within normal limits, a body mass index of 36 kg/mg^2^, and xanthomas on the third right digit and on the Achilles tendon of the right lower extremity.

**Learning Objectives**

• To consider implementing advanced novel lipid-reducing therapies, such as PCSK9, ACLY, and ANGPTL3 inhibitors, in the treatment of compound heterozygous FH patients who are not responsive to traditional medications.
• To perform genetic testing for all patients with a high degree of suspicion for FH because it can guide targeted management.
• To recognize the value of LDL-C apheresis in heterozygous FH patients unresponsive to traditional and advanced lipid-lowering therapies, who are unlikely to reach appropriate LDL-C levels for their individual risk factors.

## Past Medical History

The patient’s medical history was significant for early-onset coronary artery disease, class II obesity, hypertension, and coronary artery bypass surgery. Family history was notable for multiple first-degree relatives with early death from atherosclerotic cardiovascular disease (ASCVD) before age 40 years.

## Differential Diagnosis

The differential for this patient’s hyperlipidemia includes familial hypercholesterolemia (FH), hypothyroidism, nephrotic syndrome, iatrogenic effect, metabolic syndrome, or systemic lupus erythematosus.

## Investigations

Laboratory testing demonstrated a thyroid-stimulating hormone level of 1.61 μIU/mL (normal 0.27-4.2 μIU/mL), creatinine level of 0.71 mg/dL (normal: <1.3 mg/dL), lipoprotein a level of 27.5 nmol/L (normal: <75 nmol/L), hemoglobin A_1c_ level of 6.4% (normal: 4.0%-5.6%) and LDL-C level of 362 mg/dL. The patient’s Dutch Lipid Clinic Score without inclusion of genetic testing was 19 attributable to ASCVD, family history of severely elevated LDL-C, and early myocardial infarction and examination findings of tendon xanthomas in the patient. An adjusted score after the genetic testing was 27. Genetic testing showed 2 pathogenic mutations on exon 4 of 2 alleles of the low-density lipoprotein receptor (LDL-R) gene consistent with compound heterozygous and functionally homozygous familial hypercholesterolemia (FH). Table 1 characterizes our patient’s mutations. Doppler ultrasonography of the carotid arteries revealed moderate stenosis of the left internal carotid artery. Ankle brachial indices demonstrated peripheral arterial disease. Coronary angiography showed multivessel disease with 80% stenosis of the left main coronary artery, 100% stenosis of the left anterior descending artery, and 40% stenosis of the proximal right coronary artery. Coronary bypass grafts were patent.

**Table 1:** Genetic Testing Results

Gene	Mode of Inheritance	Variant	Zygosity	Classification
LDL-R	Autosomal semidominant	c.418 G>A p.E140K	Heterozygous	Pathogenic variant
LDL-R	Autosomal semidominant	c.337 G>A p.E113K	Heterozygous	Likely pathogenic variant

This table shows 2 independent autosomal semidominant mutations that are pathogenic in the LDL-R gene, resulting in the patient’s clinical genotype of compound heterozygous familial hypercholesterolemia.

A = adenine; G = guanine; LDL-R = low-density lipoprotein receptor.

## Management (Medical/Interventions)

**Figure 1:**
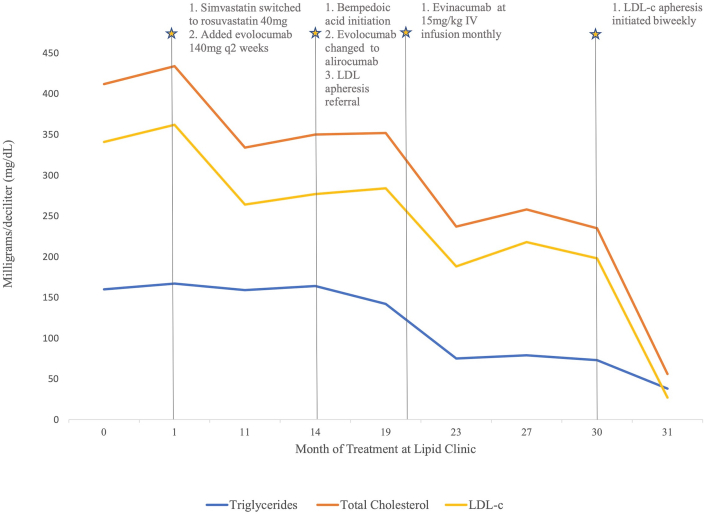
Impact of Novel Therapeutics on the Lipid Profiles of a Compound Heterozygous Familial Hypercholesterolemia Patient The patient’s lipid profile trends over the course of the treatment with the timepoints at which novel lipid-lowering therapies were implemented. There was significant LDL-C lowering with evolocumab, evinacumab, and LDL-C apheresis with an additional triglyceride-lowering effect after starting evinacumab. IV = intravenous; LDL-c = low-density lipoprotein cholesterol.

At the patient’s first clinic visit, the LDL-C level was 362 mg/dL on simvastatin and ezetimibe. The patient’s simvastatin was changed to rosuvastatin, and ezetimibe was continued. Evolocumab, a proprotein convertase subtilisin/kexin type 9 (PCSK9) inhibitor, was started at a 140-mg/mL biweekly dose and escalated to the 420 mg/3.5 mL per month dosing shortly after. Repeat lipid panels were obtained 1 to 4 months after any change in therapy. Evolocumab lowered the patient’s LDL-C from 362 to 264 mg/dL. Despite this response, the patient’s LDL-C levels remained elevated. Bempedoic acid, an adenosine triphosphate-citrate lyase (ACLY) inhibitor, was added. For insurance reasons, evolocumab 420 mg/3.5 mL monthly was switched to alirocumab 150 mg/mL every 2 weeks. Repeat blood work demonstrated no improvement. Because of the patient’s high risk for ASCVD events, evinacumab, an angiopoietin-like protein 3 (ANGPTL3) inhibitor, which is currently in clinical trials for the treatment of homozygous FH and is not commercially available, was obtained through compassionate use authorization. Mipomersen therapy was considered at this time. We discussed with the patient the known potential for hepatotoxicity of mipomersen. She expressed concern, and we decided to pursue evinacumab based on the efficacy and safety data from the ELIPSE HoFH (Evinacumab for Homozygous Familial Hypercholesterolemia) trial and clinical phase 2 trials. Following evinacumab initiation, the patient’s LDL-C decreased from 284 to 188 mg/dL. Fortunately, our institution was developing an LDL-C apheresis program, which we were able to offer to her at this point in her treatment. She was started on LDL-C apheresis therapy every 2 weeks, which consistently reduced her LDL-C to <45 mg/dL. Figure 1 demonstrates LDL-C values with the addition of each lipid-lowering therapy and LDL-C apheresis. Despite the use of 5 medication therapies, her LDL-C remained significantly elevated until she began LDL-C apheresis, which reduced her LDL-C from 188 to 27 mg/dL.

## Discussion

FH is a common autosomal dominant disease that occurs in 1 of 200 to 250 people and is often underdiagnosed, leading to early ASCVD events and reduced life expectancy. Additionally, FH treatment is often ineffective. Underdiagnosis and ineffective treatments lead to only 10% of FH patients reaching their goal LDL-C.^1^

Loss-of-function mutations in the LDL-R gene are the most common cause of FH, comprising 85% to 90% of cases.^2^ Studies demonstrate that mutations are common within the LDL-R gene, such as exon 4, the LDL ligand-binding domain. The site of our patient’s mutations can be seen in Table 1.^3^ Other causes of FH include gain-of-function mutations in the PCSK9 protein and variants of the apolipoprotein B gene. Our patient had partial function of the LDL-R, which allowed for a response to standard initial therapy of statins and ezetimibe therapy. Additionally, the utility of PCSK9 inhibitors is limited to patients with partial function of their LDL-R. Our patient, like many others with compound heterozygous FH, had extensive coronary artery disease, which required the addition of therapies while her LDL-C was >70 mg/dL.^1^ Many novel LDL lipid-lowering therapies include ANGPTL3 inhibitors, small interfering RNA against PCSK9, microsomal triglyceride transfer proteins, and apolipoprotein B inhibitors.^4^ There is minimal guidance on how to approach the integration of recently approved lipid-lowering medications into the care of patients with refractory FH. We describe the stepwise implementation of multiple novel LDL-C–lowering medications, including evolocumab and alirocumab (PCSK9 inhibitors), bempedoic acid (ACLY inhibitor), and evinacumab (ANGPTL3 inhibitor), in conjunction with statin and ezetimibe therapy.

In our case, we used repeat lipid profiles at 1- to 4-month intervals to monitor the response to therapeutic interventions, including newer agents such as bempedoic acid and evinacumab. Additionally, the clinical case showed a partial therapeutic response in a compound heterozygous FH patient to evinacumab therapy, which has been approved for only homozygous FH patients. Interestingly, we noted a 50% decrease in triglycerides with evinacumab therapy, which is not surprising given the role of ANGPTL3 in inhibiting triglyceride breakdown.^5^^,^^6^ Ultimately, our patient required LDL-C apheresis, for which she sustained an excellent response, too. LDL-C apheresis is typically recommended for functional heterozygous patients with an LDL-C level of >160 mg/dL and very high-risk characteristics, such as established ASCVD.^7^

## Follow-Up

In spite of 5 lipid-lowering agents, the patient had persistently elevated LDL-C and required LDL-C apheresis. Evinacumab was discontinued after starting LDL-C apheresis. Additionally, the patient was treated with semaglutide, a glucagon-like peptide 1 receptor agonist, to reduce her ASCVD risk and to promote weight loss.

## Conclusions

We describe a case of a compound heterozygous FH patient refractory to current advanced LDL-C–lowering therapies. We demonstrate the impact of novel therapeutics, including bempedoic acid, evolocumab, alirocumab, and evinacumab, when implemented through a stepwise approach. Notably, we highlight the continued clinical utility of LDL-C apheresis despite novel LDL-C–lowering medications in the treatment of a compound heterozygous FH patient with 2 different LDL-R mutations.

## Funding Support and Author Disclosures

The authors have reported that they have no relationships relevant to the contents of this paper to disclose.
